# A rare case of parapelvic cyst: A case report

**DOI:** 10.1016/j.radcr.2023.10.025

**Published:** 2023-11-02

**Authors:** Leni Santiana, Adi Maulana Samsudin

**Affiliations:** Department of Radiology, Faculty of Medicine, University of Padjadjaran, Dr. Hasan Sadikin General Hospital, Jl. Pasteur No.38, Pasteur, Sukajadi, Bandung City, West Java 40161 Indonesia

**Keywords:** Parapelvic cyst, Ultrasonography, Computed-tomography scan

## Abstract

Parapelvic cyst (PPC) is a rare cyst that arises from the renal parenchyma and usually does not cause symptoms. However, PPC may cause compression of the pelvicalyceal system resulting in obstructive symptoms. Ultrasonography may not be sufficient to diagnose PPC since they are often misdiagnosed with hydronephrosis, thus computed tomography of the renal is required. In this case, we present, a 27 years old male with a 3 years history of partial urinary retention presented with intermittent loin pain. Hydronephrosis was found on ultrasonography but failed to identify the source of obstruction. A CT scan was conducted and a PPC was confirmed. Nephrostomy was scheduled for ureteral dilation and the patient responded well 4 months after surgery. In this case, we highlight the role of radiology in identifying and differentiating the PPC.

## Introduction

Renal cyst, including pararenal cyst, is the most common lesion found in the kidney. Renal cysts affect approximately 40% of all individuals undergoing renal imaging that can be unilateral/bilateral, focal/multifocal, or acquired/congenital [1]. Cysts in the kidney's hilus that are closely linked to the renal pelvis and calyces are referred to as parapelvic cysts (PPC) of the kidney. PPC is different from simple renal pelvis such that PPC does not arise from the renal parenchyma, but it is contiguous to the renal pelvis but does not communicate with the collecting system. It may compress and displace the renal pelvis if it is large enough [2]. At least 20% of adults will have formed simple renal cysts by the age of 40 and up to 33% will have developed renal cysts at the age of 60. Most PPCs are found by chance and are generally symptomless but as they are closely associated with the hilar vessels and the collecting system they can produce symptoms of renal obstruction. On ultrasound, PPCs are often misdiagnosed as hydronephrosis since they produce hypoechoic lesions within the renal pelvis. Thus, a CT scan with contrast is necessary to differentiate them [3].

Several studies have demonstrated the imaging of PPC. A PPC at times may cause compression of the pelvicalyceal system resulting in hydronephrosis. Commonly, PPCs do not communicate with the collecting system and are believed to be lymphatic in origin. A PPC is a nongenetic cyst with pathological changes; the incidence is about 1%-2% of total renal cysts [4]. In this case, we present a rare case of a PPC-causing hydronephrosis. We highlight the role of radiology in identifying the cyst.

## Case

**Fig. 1:**
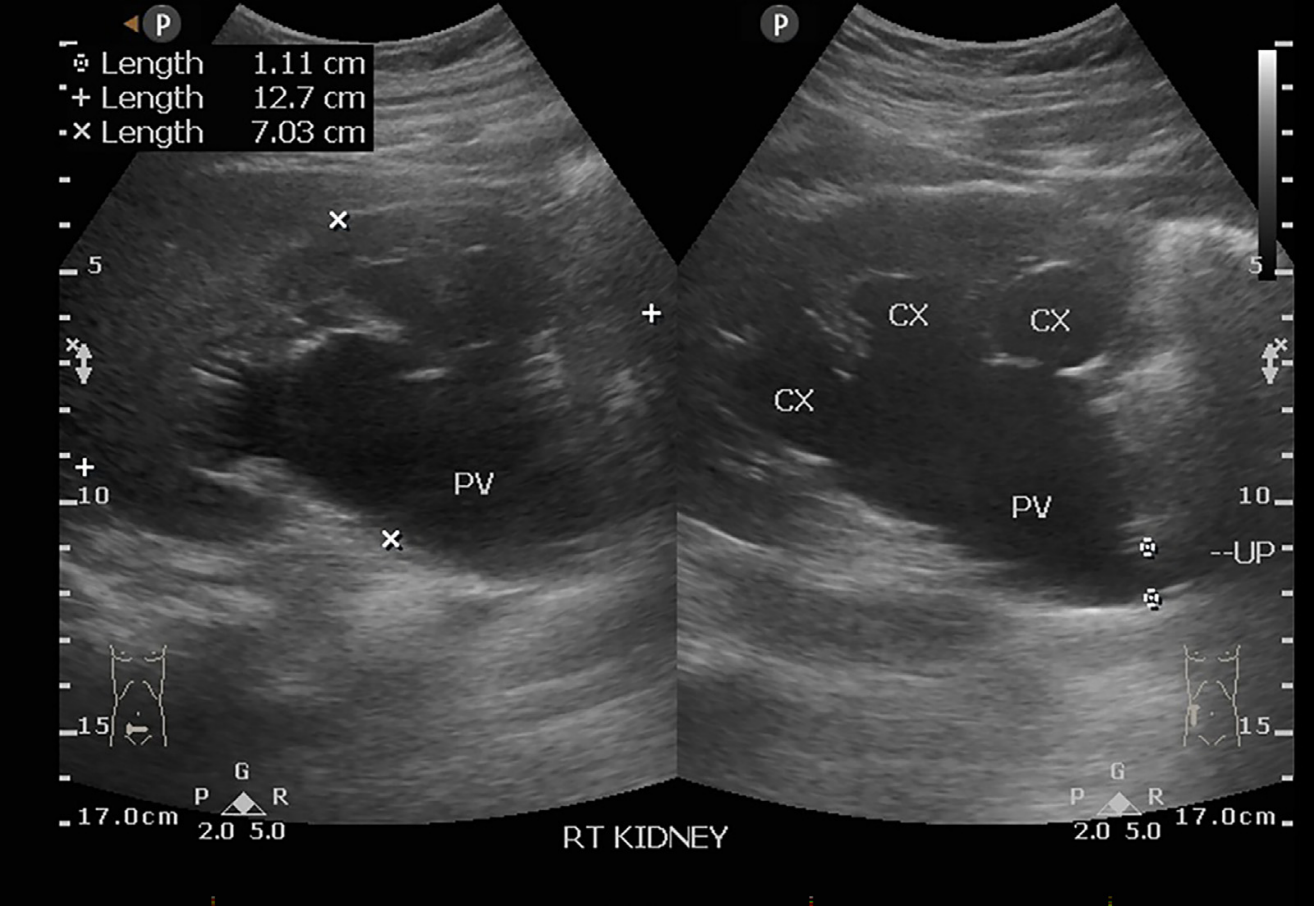
Longitudinal ultrasound of the right kidney demonstrated pelvicalyceal dilatation and a dilated proximal ureter. CX, calyx; PV, Pelvis.

A 27-year-old male with a 3-year history of partial urinary retention presented with intermittent loin pain. History of fever, stone passage through urine, and hematuria was denied. His vital signs were within normal limits. Physical examinations were within normal limits. Laboratory examinations including hematology, renal function, and urinalysis found no abnormalities. Initial ultrasonography was performed and found pelvicalyceal dilatation and the proximal ureter was dilated, indicating pelvico ureteric junction obstruction (PUJO) (Fig. 1).

**Fig. 2:**
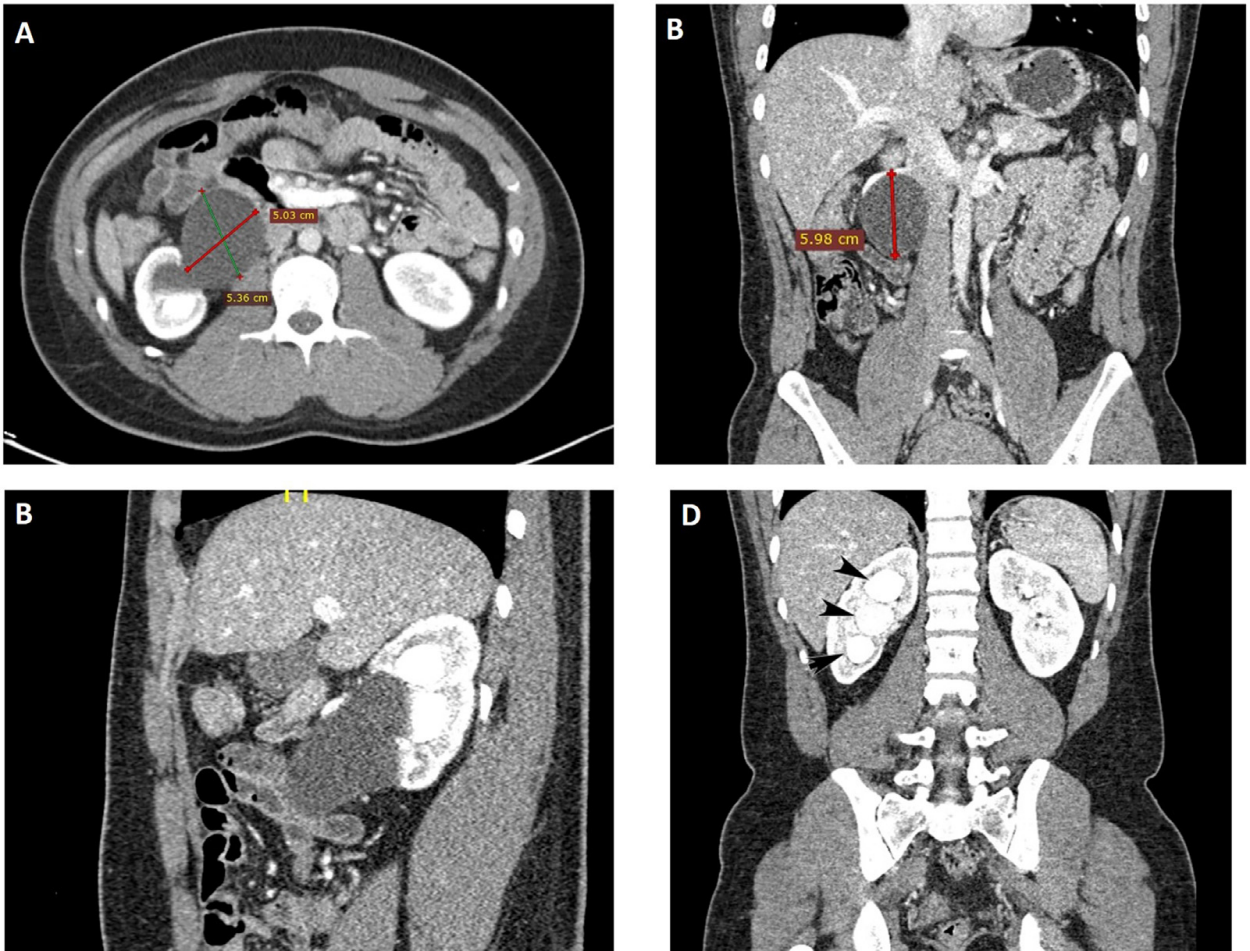
Contrast CT scan demonstrated the PPC with hypodense lesion (A) Transverse view with a dimension of the cyst measuring 5.03 cm (red line) x 5.36 cm (green line), (B) Coronal view with dimension of the cyst measuring 5.98 cm (red line), (C) sagittal view, (D) coronal view Right calyceal dilatation indicating obstruction.

Renal ultrasound was unable to identify the source of obstruction, thus further imaging was conducted. A renal computed tomography (CT) CT scan was performed and showed the normal size of the right kidney measuring 10.44 × 4.68 cm. The Parenchyma structure is homogeneous, pelvocalyceal system is widened. There was a hypodense lesion in the renal pelvis, extending to the proximal ureter that was not filled with contrast measuring 5.03 × 5.36 × 5.98 cm (Fig. 2).

The proximal right ureter was dilated. The cyst presses the right renal pelvic system and right proximal ureter causing PUJO, causing hydronephrosis grade III. He was advised to undergo a nephrostomy for ureteral dilation followed by double J stent placement. A percutaneous approach for cyst removal was deemed very difficult in this patient, thus nephrostomy was conducted using an 8-8.9 Fr nephroscope. To be able to remove the cyst, the cyst wall had to be very thin and well-visualized to avoid renal parenchyma injury. However, in our case, the cyst wall was too thick and poorly visualized, thus cyst removal was not conducted. Cyst removal failed, thus we proceeded to perform ureteral dilatation. The double J stent was inserted and bleeding was checked. Four months after surgery, the cyst remained, but the patient was asymptomatic.

## Discussion

PPC is a relatively rare disease with an incidence of 1%-2% of total renal cysts. PPCs do not communicate with the collecting system and are believed to be lymphatic in origin. While other evidence suggests that PPCs are embryological remnants of the developing mesonephric duct. The mechanism of formation and tissue structure of PPC are almost the same as the simple kidney cyst. Generally, this disease is either caused as a result of congenital dysplasia or acquired obstruction in the kidney. The resulting anatomical disturbance in patients with PPC may cause chronic inflammation and local expansion of pelvic lymphatic vessels. This mechanism was thought to be responsible for the associated history of urinary tract obstruction and infection in patients with PPC. The association between PPC with other anatomical anomalies remained unknown. However, it is possible that the associated anomaly within the urinary tract contributes to UTI which may increase the risk of PPC [5].

Marret et al found that the histology within the PPC consists of a single flat epithelial cell layer, suggesting that PPC and the urinary tract are structures of independent origins. A communication between the cyst and the collecting system has never been shown in the literature. They are usually asymptomatic unless the cyst obstructs the ureter, causing PUJO. Due to its rarity, there are only a few studies reporting the case of hydronephrosis due to PPC. In this case, we highlight the role of radiographic imaging in PPC.

Most renal cysts are symptomless, but they can occasionally cause lumbar discomfort, hematuria, hypertension, and hydronephrosis due to obstruction by the cyst. However, the possibility of a PPC must be kept in mind when the asymptomatic hydronephrosis is noted by ultrasonography with unknown causative obstructive lesion, since PPCs are often misdiagnosed as hydronephrosis. In our case, the patient reported intermittent loin pain without significant urinary tract symptoms due to the hydronephrosis caused by the cyst [6].

Only a few studies reported the radiologic features of PPC. PPC can mimic hydronephrosis and so can be confused with pelvi-ureteric junction obstruction (PUJO) as the ureter in both conditions is not dilated. This could be well differentiated at the excretory phase of the CT-IVP study (CT urography) that demonstrates stretched (but not dilated) collecting system by the cyst in contrary to PUJO at which the contrast will be pooled within the dilated collecting system. It can cause pelvicalyceal system dilatation by its mass effect [7]. On IVU, PPC can show stretching and compression of calyces, similar to the appearance of renal sinus lipomatosis which involves proliferation of sinus fat leading to a mass effect on the intrarenal collecting system. There will be no contrast enhancement following the IV contrast administration nor hydroureter upon the CT scan. Those renal cysts are usually centrally placed on ultrasounds, and they may be misinterpreted as hydronephrosis. Therefore, for differential diagnosis, ultrasound alone may lead to misdiagnosis, particularly when there is a discordance between a major dilatation and good drainage on renal scan; therefore, a complete imaging work-up with MRI or CT-scan and retrograde pyelography is essential. In our case, the cyst was not filled with contrast, which differentiates it from hydronephrosis and renal cysts, both of which are usually filled with contrast.

A case report by Mao et al. [8] reported PPC was misdiagnosed as hydronephrosis. Ultrasound revealed a hypoechoic area in the right central renal sinus, suggesting right hydronephrosis, but the obstructive site could not be found. For further evaluation of the obstructive site for the hydronephrosis, they arranged a CT scan and revealed a PPC with a mild displacement of the collecting system without hydronephrosis. Since the patient had no symptoms, no more treatment was given and the patient was discharged. Mao et al. [8] highlighted the importance of CT scan in differentiating PPC from hydronephrosis.

Symptomatic or complicated PPC mandates corrective intervention. Various treatments for simple renal cysts have been proposed with varying outcomes, including sclero- therapy, laparoscopic unroofing, and percutaneous ablation. Compared with the treatment of simple renal cysts, the treatment of PPC can be more difficult because of the location near the renal hilum and renal vessels. Sclerotherapy is considered to be contraindicated in the treatment of PPC because it can provoke perirenal inflammation and subsequently, ureteropelvic junction obstruction. Percutaneous aspiration of PPC has been condemned in view of grave complications like retroperitoneal leakage of sclerosant and perirenal inflammation and the recurrence rate is 22.8%-30%. Percutaneous nephroscopy-guided resection and retrograde ureteroscopic resection are widely accepted treatment options although they may be limited by the size and location of the cyst. The work access sites connected to percutaneous nephrostomy invariably result in renal injury, such as severe perinephritis, retroperitoneal abscess, and subsequent ureteropelvic junction obstruction. This is in contrast to percutaneous resection or ablation. For some cysts, the retrograde method might be less invasive. The danger of severe consequences has decreased because of the flexible ureteroscopic approach. One of the most important aspects of PPC removal is the placement of the renal cyst wall. PPC wall thickness may determine if it is removable or not. Laparoscopic management has been sparsely reported and apprehended in literature because of extensive dissection and technical difficulty [9]. In this case, ureteral dilatation followed by a DJ stent was inserted for at least 4 months and resulted in a good outcome. PPC removal was not conducted since it was deemed risky for bleeding, had a thick PPC wall, and poor visualization of the cyst entry during surgery. This patient was an example of how important contrast CT is in identifying the source of PUJO.

## Conclusion

PPC is usually asymptomatic, however, if it obstructs the normal urinary tract, it will cause hydronephrosis and comprehensive imaging is needed to better visualize the cyst. This case represents one example of a PPC causing hydronephrosis with a good prognosis even without cyst removal surgery.

## Patient consent

I, on behalf of all authors, confirm that we have obtained written informed consent for publication from the patient. Subjects voluntarily participate in this study. Subject agrees that Subject has been given the opportunity to ask questions and have them answered to Subject's satisfaction. The subject had noticed that his ultrasound and CT scan findings would be published for scientific purposes. Subject has received a copy of this consent form signed by the researcher.
